# Aptamer-Based Gold Nanoparticle Lateral Flow Assay for Rapid Detection of Cardiac Troponin I

**DOI:** 10.3390/bios15120776

**Published:** 2025-11-26

**Authors:** Jing Zhang, Jiayi Pang, Cheng Cui

**Affiliations:** 1 School of Biological Science and Medical Engineering, Hunan University of Technology, Zhuzhou 412007, China; 23408270123@stu.hut.edu.cn; 2 Molecular Science and Biomedicine Laboratory (MBL), State Key Laboratory of Chemo and Biosensing, College of Chemistry and Chemical Engineering, College of Biology, Aptamer Engineering Center of Hunan Province, Hunan University, Changsha 410082, China

**Keywords:** cTnI, aptamer, gold nanoparticle, lateral flow, acute myocardial infarction, rapid detection, sandwich

## Abstract

Cardiac troponin I (cTnI) is a critical biomarker for the diagnosis of acute myocardial infarction (AMI), but conventional detection methods are often time-consuming and require specialized laboratory equipment. To meet the need for rapid and feasible detection, there is an urgent demand for methods that are fast, specific, and easy to use. In this study, two aptamers (Tro4 and Tro6), which specifically bind to different epitopes of cTnI, were employed to construct a dual-aptamer sandwich system on a lateral flow assay (LFA) strip. The test strip can deliver results within 10 min and shows a detection limit of 11.70 ng·mL^−1^. It also exhibited excellent stability after storage at room temperature for up to four months. The assay demonstrated high analytical accuracy, as evidenced by recovery rates from spiked serum samples ranging from 95.11% to 103.17%. These results suggest that the proposed aptamer-based LFA is highly suitable for rapid screening of cTnI, especially in point-of-care settings and resource-limited environments. From a diagnostic perspective, this method holds great promise for improving the timely detection and management of AMI and other myocardial injuries.

## 1. Introduction

Cardiovascular diseases remain the leading cause of death worldwide, among which acute myocardial infarction (AMI) accounts for the highest mortality rate [1]. AMI is caused by acute and sustained ischemia and hypoxia of the coronary arteries, leading to irreversible myocardial necrosis. Diagnosis typically relies on clinical symptoms such as chest pain, electrocardiogram (ECG) abnormalities, and the detection of cardiac biomarkers in body fluids [2]. Among these, measuring cardiac biomarkers in blood or serum has proven to be a highly accurate and reliable method. Cardiac troponin I (cTnI), in particular, is a highly sensitive and specific marker for myocardial injury and is widely regarded as the gold standard for AMI diagnosis [3,4]. In healthy individuals, the baseline serum concentration of cTnI ranges from 0.4 to 2.0 ng·mL^−1^ [5,6], while in AMI patients, it can increase dramatically to 100–300 ng·mL^−1^ [7,8]. Typically, cTnI levels rise within 3–4 h after the onset of myocardial injury, peak at 12–24 h, and return to baseline within 6–8 days [9,10]. Given the narrow diagnostic window and rapid progression of AMI, the development of rapid and accessible detection methods for cTnI is of great clinical importance.

In recent years, various innovative methods for the detection of cTnI have been extensively investigated, including colorimetric assays [11], fluorescence-based techniques [12], chemiluminescence immunoassays [13,14], electrochemical sensors [15], and surface plasmon resonance biosensors [16]. Although these methods offer excellent sensitivity and quantitative accuracy, their clinical application is limited by labor-intensive procedures, long assay times, and dependence on sophisticated instruments and trained personnel. As such, they are not well-suited for point-of-care testing (POCT) or use in decentralized settings.

Lateral flow assays (LFAs) have emerged as powerful diagnostic tools for on-site detection of various biomarkers due to their rapid response, user-friendly operation, low cost, and ability to yield visually interpretable results without the need for specialized equipment [17]. Among them, gold nanoparticle (AuNP)-based LFAs have been widely adopted in POCT owing to their strong optical signal, excellent biocompatibility, and high specificity [18]. Rapid identification of AMI through POCT, followed by timely pre-hospital intervention by community clinics or emergency medical teams, can significantly improve the management of acute chest pain and enhance patient survival and prognosis [19]. Antibody-based AuNP-LFAs targeting cTnI have already shown promising performance in the early diagnosis of AMI [20]. However, the inherent limitations of antibodies—including batch-to-batch variability, high production cost, low thermal stability, and restricted chemical modifiability—have hindered their broader application in LFA platforms.

Aptamers, which are single-stranded oligonucleotides capable of binding to specific targets with high affinity and selectivity, offer several advantages over conventional antibodies. These include lower production cost, greater thermal and chemical stability, and ease of functional modification. Consequently, aptamers have been proposed as promising substitutes for antibodies in biosensing applications [21]. Their integration into LFA platforms has demonstrated favorable analytical performance across various target analytes [22]. Recent studies have also explored aptamer-based LFAs for the detection of myocardial infarction biomarkers. For instance, Bachu et al. developed an electrochemiluminescence-based LFA on a microfluidic hybrid platform for the detection of cardiac troponin T [23]. This method employed aptamers and screen-printed electrodes to facilitate signal transduction under an applied direct current voltage. Although the assay exhibited high sensitivity, its reliance on external power sources and specialized imaging equipment may limit its practical applicability in resource-limited or emergency settings.

**Scheme 1:**
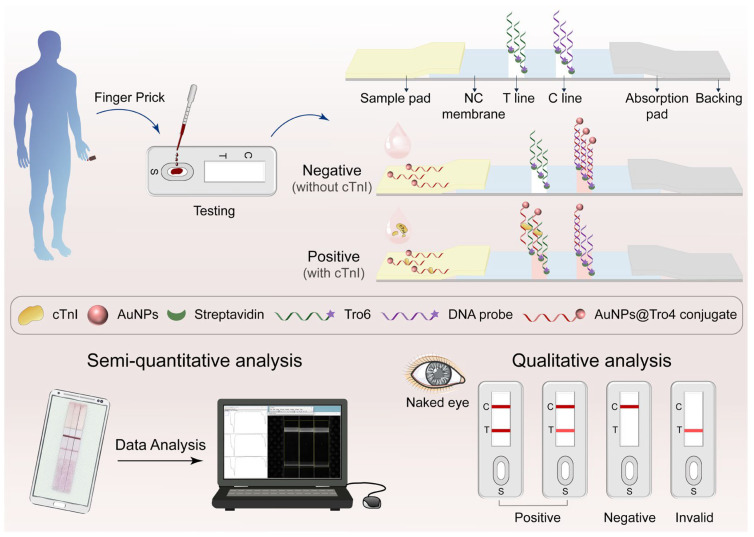
Schematic illustration of the aptamer-based LFA for cTnI detection. Upon finger-prick blood sampling, the sample is applied to the sample pad and migrates through the strip via capillary action. In the presence of cTnI, AuNP@Tro4 conjugates bind to the target and form AuNP@Tro4–cTnI complexes, which are subsequently captured by Tro6 immobilized at the T line, producing a visible red band due to AuNP accumulation. In contrast, in the absence of cTnI, no sandwich complex is formed and no signal appears at the T line. Regardless of cTnI presence, the C line contains a DNA probe that captures unbound AuNP@Tro4 conjugates, serving as an internal control to verify assay validity. The results can be interpreted qualitatively by the naked eye or semi-quantitatively via digital analysis of strip images.

Given the urgent clinical need for rapid, accessible and equipment-free cTnI testing, we propose a visual dual-aptamer sandwich-type LFA incorporating AuNPs for the rapid and sensitive detection of cTnI, as illustrated in Scheme 1. The assay is based on the specific affinity between cTnI and two selected DNA aptamers (Tro4 and Tro6), which together form a sandwich complex (Tro4–cTnI–Tro6). In the presence of cTnI, the AuNP@Tro4 conjugates bind to the target protein to form AuNPs@Tro4–cTnI complexes. These complexes migrate along the nitrocellulose (NC) membrane via capillary action and are subsequently captured by Tro6 immobilized at the test (T) line, generating a visible signal due to the accumulation of AuNPs. Conversely, in the absence of cTnI, no sandwich complex is formed, and thus no color develops at the T line. Regardless of the presence of cTnI, the DNA probe immobilized at the control (C) line captures the excess AuNPs@Tro4 conjugates to validate the assay. This platform combines the high specificity of aptamer recognition with the signal amplification properties of AuNPs, offering a robust, cost-effective, and field-deployable tool for early diagnosis of AMI and monitoring of cardiovascular diseases.

## 2. Materials and Methods

### 2.1. General Materials

All reagents were used as received without further purification. Unless otherwise specified, reagents were purchased from Thermo Fisher Scientific (Waltham, MA, USA). Bovine serum albumin (BSA) was obtained from Beyotime Biotechnology (Shanghai, China). Tris-EDTA (TE) buffer, Triton X-100, and Tween-20 were purchased from Sangon Biotech (Shanghai, China). Streptavidin (SA) and potassium carbonate (K_2_CO_3_) were obtained from Beijing Solarbio Science & Technology (Beijing, China). Fetal bovine serum (FBS) was purchased from Zeta Life (Xi’an, China). Magnesium Dichloride (MgCl_2_) was obtained from Energy Chemical (Shanghai, China). 96-well plates were purchased from NEST Biotechnology (Wuxi, China). NC membrane and Amicon Ultra-0.5 centrifugal filter unit with Ultracel-30 membrane were purchased from Merck Millipore (Darmstadt, Germany). Absorbent pads, polyvinyl chloride (PVC) backing cards, and sample pads were obtained from Jiening Biotech (Shanghai, China). The DNA sequences listed in Table 1 were synthesized by Sangon Biotech (Shanghai, China). Ultrapure water used in all experiments was prepared using a Milli-Q Biocel system (Millipore, Burlington, MA, USA). A smartphone (iPhone 14, running iOS 14) was used for image capture, and image analysis was performed using ImageJ 1.8.0 software (National Institutes of Health, Bethesda, MD, USA). The BioDot XYZ dispensing platform (Model XYZ3010) for LFA strip fabrication was purchased from Shanghai Kinbio Technology (Shanghai, China).

### 2.2. Synthesis, Conjugation, and Characterization of AuNPs@Tro4

AuNPs were synthesized via the classical citrate reduction method. Briefly, 0.5 mL of HAuCl_4_ solution (1%, *w*/*v*) was added to 49.5 mL of ultrapure water under vigorous stirring and heated to boiling. Upon reaching the boiling point, 2 mL of trisodium citrate solution (1%, *w*/*v*) was rapidly added. A gradual color change in the solution was observed, indicating the formation of AuNPs. The reaction mixture was kept boiling under constant conditions for an additional 10 min after the color stabilized. The resulting colloidal solution was then cooled to room temperature (RT) in the dark and adjusted to a final volume of 100 mL with ultrapure water. The AuNPs solution was stored at 4 °C in the dark for further use.

To prepare AuNPs@Tro4 conjugates, thiol-modified DNA aptamer Tro4 was attached to the AuNPs via gold–thiol interactions. Before conjugation, 1 mL of freshly prepared AuNPs solution was centrifuged at 12,000 rpm for 15 min, and the pellet was resuspended in 0.25 mL of ultrapure water. In parallel, the thiol-modified aptamer probe (50 μM, 30 μL) was incubated with tris(2-carboxyethyl)phosphine (TCEP, 50 μM, 30 μL) for 2 h to activate the thiol groups. The reduced aptamer was then mixed with the AuNPs solution, followed by the addition of 4.5 μL trisodium citrate (500 mM, pH 3.0). The mixture was incubated at RT for 1 h. Subsequently, 12 μL of NaCl solution (1 M) was added, and incubation continued for another hour at RT. Then, 60 μL of HEPES buffer (500 mM, pH 7.6) was introduced to adjust the pH to alkaline conditions. The mixture was further incubated at RT for 2 h, followed by blocking with 10% BSA to reduce nonspecific binding. The AuNPs@Tro4 conjugates were collected by centrifugation at 12,000 rpm for 15 min, washed three times with Tris-HCl buffer (10 mM, pH 7.0), and finally resuspended in 100 μL of the same buffer for subsequent use. The successful conjugation of aptamer to AuNPs was confirmed by UV–visible spectrophotometry.

### 2.3. Preparation and Assembly of Aptamer-Based LFA

LFA was designed based on a dual-aptamer sandwich principle. The cTnI-specific aptamers, designated Tro4 and Tro6, were adopted from previously reported sequences and have been verified to form a stable sandwich structure [24,25]. The preparation and assembly of the LFA strip were performed according to procedures described in our previous work, with minor modifications [26]. Specifically, the sample pads and NC membranes were pretreated before assembly. The sample pads were saturated with 10 mM phosphate-buffered saline (PBS) containing 0.1% (*v*/*v*) Tween-20 and 1% (*w*/*v*) BSA for 30 min and subsequently dried overnight at RT. For the T line, streptavidin-conjugated Tro6 (1 OD/30 μL) was immobilized on the NC membrane. For the C line, a streptavidin-conjugated DNA probe (1 OD/30 μL) was similarly immobilized. The membranes were then dried at 37 °C for 30 min. Following this, the NC membrane, sample pad, and absorbent pad were sequentially assembled onto a PVC backing card. The assembled sheet was then cut into strips of 4 mm width and stored at RT in sealed foil pouches until use.

### 2.4. Aptamer-Based LFAs for cTnI Detection

The LFA procedure was performed as follows. A 100 μL reaction mixture containing AuNPs–Tro4 conjugates, the sample, 10 mM Tris-HCl buffer, and 1% (*v*/*v*) Tween-20 was prepared in the wells of a 96-well ELISA plate. Subsequently, the assembled LFA strip was vertically inserted into the well and allowed to react at RT for 10 min. The solution migrated along the NC membrane by capillary action. For the detection of cTnI in serum samples, artificial serum was spiked with various concentrations of cTnI and diluted 20-fold in PBS. The spiked samples were then added to the reaction mixture under identical assay conditions. The visible signals on the T and C lines were captured using a smartphone camera, and the signal intensities were quantitatively analyzed using ImageJ software. Each sample was tested in triplicate under the same conditions.

## 3. Results and Discussion

### 3.1. Characterization of AuNPs and AuNPs@Tro4

**Figure 1:**
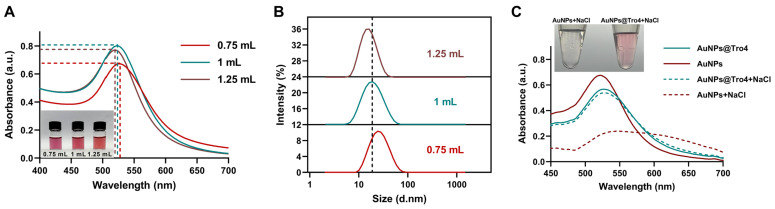
Characterization of AuNPs and AuNPs@Tro4. (**A**) UV–vis spectra of AuNPs prepared using different volumes of trisodium citrate (0.75, 1.0, and 1.25 mL). Inset: corresponding color of the colloidal solutions. (**B**) DLS analysis of AuNPs synthesized with varying volumes of trisodium citrate (0.75, 1.0, and 1.25 mL). (**C**) UV–vis spectra of AuNPs and AuNPs@Tro4 before and after addition of 1 M NaCl.

AuNPs were synthesized via the trisodium citrate reduction method, where the particle size is inversely correlated with the amount of trisodium citrate added. As shown in Figure 1A, UV–visible spectrophotometry was used to characterize AuNPs synthesized with different volumes of trisodium citrate. When the volume of trisodium citrate was decreased from 1.25 mL to 0.75 mL, the color of the AuNPs solution gradually shifted toward purple, indicating an increase in particle size. Correspondingly, the maximum absorbance wavelength showed a gradual red shift, and the absorption peaks became broader. This suggests that larger AuNPs exhibit more stable and uniform size distributions.

To further confirm the particle size changes, dynamic light scattering (DLS) analysis was performed (Figure 1B). The average hydrodynamic diameters of AuNPs synthesized with 1.25, 1.0, and 0.75 mL trisodium citrate were approximately 13.5 nm, 17.2 nm, and 22.4 nm, respectively. These results clearly demonstrate that lower citrate volumes result in larger nanoparticle sizes, which is consistent with the color change and red-shifted UV–vis spectra observed in Figure 1A.

Successful conjugation between AuNPs and the thiolated aptamer Tro4 is a critical step in ensuring the stability and reliability of the LFA system. As shown in Figure 1C, the UV–vis spectrum of unconjugated AuNPs displayed a sharp absorbance peak at 520 nm (red solid line), whereas the AuNPs@Tro4 conjugates exhibited a red-shifted peak at 527 nm (green solid line), indicating increased particle size due to surface modification and confirming successful aptamer conjugation. Additionally, bare AuNPs and AuNPs@Tro4 were analyzed by DLS analysis, which revealed a significant increase in hydrodynamic diameter upon Tro4 conjugation (Figure S1). To further assess the stability of the conjugates, 1 M NaCl was added to both AuNPs and AuNPs@Tro4 solutions. Upon salt addition, the unconjugated AuNPs aggregated severely, as evidenced by the loss of a defined absorbance peak and the appearance of a flattened, broadened spectrum (Figure 1C, red dashed line). In contrast, the AuNPs@Tro4 conjugates retained a distinct absorption peak with minimal shift (Figure 1C, green dashed line), demonstrating strong resistance to salt-induced aggregation. These results confirm that the aptamer forms a protective layer around the AuNPs, maintaining their colloidal stability and significantly improving their resistance to nonspecific interference, which is essential for reliable lateral flow assay performance.

### 3.2. Optimization of Detection Parameters

To achieve high sensitivity and minimize background interference in the detection of cTnI, several critical experimental parameters were systematically optimized. These included the amount of trisodium citrate used in AuNP synthesis, the concentrations of DNA probe and Tro6 on the C and T lines, the concentration of Tro4 for conjugation, as well as the composition of the sample buffer, including buffer type, surfactant type, and surfactant concentration.

**Figure 2:**
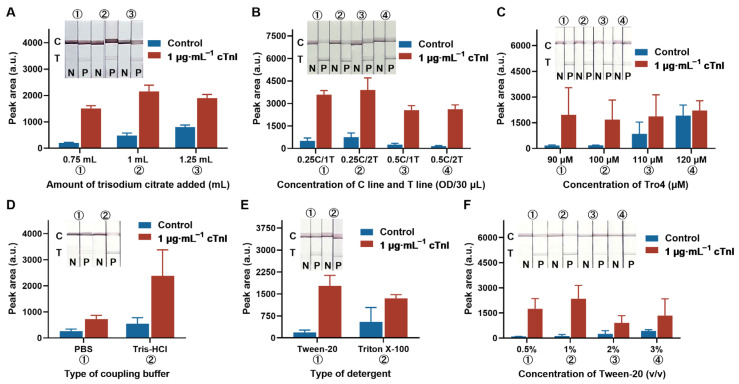
Optimization of detection parameters for the aptamer-based LFA. (**A**) Effect of trisodium citrate volume (0.75, 1.0, and 1.25 mL) on the detection of 1 μg·mL^−1^ cTnI. (**B**) Effect of C-line and T-line probe concentrations (0.25C/1T, 0.25C/2T, 0.5C/1T, and 0.5C/2T, OD/30 μL). (**C**) Effect of Tro4 concentration (90, 100, 110, and 120 μM). (**D**) Effect of coupling buffer type (PBS and Tris-HCl). (**E**) Effect of detergent type (Tween-20 and Triton X-100). (**F**) Effect of Tween-20 concentration (0.5%, 1%, 2%, and 3%, *v*/*v*). Control and test groups represent blank and 1 μg·mL^−1^ cTnI, respectively. Peak areas were measured from T-line intensities.

AuNPs were employed as the signal transducers in the LFA. Their size, which can be controlled by the amount of trisodium citrate added during synthesis, significantly affects the optical signal and assay background. As shown in Figure 2A, although all three AuNP preparations produced visible signals at the T line, the AuNPs synthesized with 0.75 mL trisodium citrate generated the lowest nonspecific signal, resulting in a better signal-to-noise ratio. Therefore, this volume was selected for subsequent AuNP synthesis.

The relative concentrations of the capture probes at the C and T lines also play a key role in ensuring detection accuracy. Excessive C-line probe concentration may excessively retain AuNPs, thereby reducing the T-line signal and wasting reagents, while overly high T-line probe concentration may result in nonspecific adsorption. As shown in Figure 2B, orthogonal experiments tested combinations of 0.25 and 0.5 OD/30 µL for the C-line probe and 1 and 2 OD/30 µL for Tro6 on the T line. Increasing T-line probe concentration enhanced the peak area. The 0.5C/2T group exhibited optimal performance, yielding uniform signals with minimal background noise and reduced false positives in the blank group. Thus, the optimal probe concentrations were set as 0.5 OD/30 µL for the C line and 2 OD/30 µL for the T line.

The concentration of aptamer Tro4 used for AuNP conjugation is another critical factor influencing both sensitivity and specificity. Insufficient Tro4 may lead to weak signals or aggregation of nanoparticles, while excessive Tro4 can promote nonspecific binding and background interference. As shown in Figure 2C, the highest peak areas were observed at 110 and 120 µM, but the control groups at these concentrations exhibited increased false positives. Comparatively, 90 µM Tro4 provided a more stable signal with low background, and was therefore selected as the optimal conjugation concentration, balancing cost, signal strength, and specificity.

Buffer composition affects the conformational integrity of aptamers and their binding affinity to targets. PBS (pH 7.4) and Tris-HCl (pH 8.0) were compared to identify the optimal buffer system. As shown in Figure 2D, strips using Tris-HCl exhibited stronger T-line signal and larger peak areas than those using PBS, likely due to its favorable pH supporting cTnI stability and aptamer affinity. Hence, 10 mM Tris-HCl (pH 8.0) was chosen as the sample buffer. Surfactants are essential for controlling fluid flow and reducing nonspecific adsorption in LFAs. Two commonly used non-ionic surfactants, Tween-20 and Triton X-100, were compared. As shown in Figure 2E, 1% Tween-20 provided better T-line signal and lower background than Triton X-100. Further optimization of Tween-20 concentration (0.5% to 3.0% *v*/*v*) revealed that 1.0% offered the best balance between signal strength and specificity, while higher concentrations increased false positives and reduced clarity (Figure 2F).

Collectively, these optimizations enhanced the assay’s overall performance, providing a reliable basis for subsequent analytical evaluation.

### 3.3. Analytical Performance of the Proposed LFA

#### 3.3.1. Sensitivity

**Figure 3:**
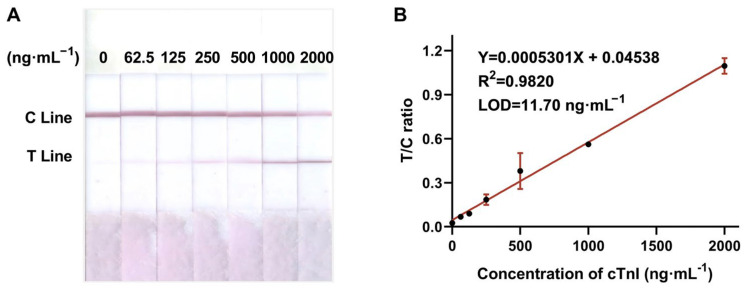
Sensitivity evaluation of the aptamer-based LFA for cTnI detection. (**A**) Visual results of the LFA strips after applying cTnI at different concentrations (0, 62.5, 125, 250, 500, 1000, and 2000 ng·mL^−1^). (**B**) Calibration curve of the T/C ratio versus cTnI concentration in the range of 0–2000 ng·mL^−1^. All values are presented as mean ± standard deviation (SD), n = 3.

Under optimized experimental conditions, the proposed aptamer-based LFA was evaluated for its sensitivity in detecting gradient concentrations of cTnI (0, 62.5, 125, 250, 500, 1000, and 2000 ng·mL^−1^). As shown in Figure 3A, the color intensity of the T line gradually increased with rising cTnI concentrations, indicating successful accumulation of AuNPs-Tro4 complexes. Notably, even at a concentration of 62.5 ng·mL^−1^, the T line was clearly distinguishable from the negative control, demonstrating high visual sensitivity. The corresponding T/C ratios exhibited a good linear relationship with cTnI concentration in the range of 0–2000 ng·mL^−1^, with the regression equation y = 0.0005301x + 0.04538 and a correlation coefficient R^2^ = 0.9820 (Figure 3B). Based on the 3σ rule, the limit of detection (LOD) was calculated to be 11.70 ng·mL^−1^, where σ represents the standard deviation of the blank group. This LOD is significantly lower than that reported value for creatine kinase-MB detection using a similar aptamer-based LFA approach (LOD = 3.6 μg·mL^−1^) [27], and it meets the clinical sensitivity requirements for cTnI quantification [7,8].

The obtained results herein were compared with those of other representative aptamer-based LFA sensors targeting different analytes, as well as various types of cTnI detection sensors (Table 2). The findings indicate that, compared to other aptamer-based LFAs employing AuNPs as signal reporters, the proposed method exhibits competitive sensitivity. Although the LOD is higher than that of some electrochemiluminescence (ECL) or electrochemical aptasensors developed for cTnI, the present assay offers faster detection speed and simplified operational procedures, enabling effective detection without the need for complex instrumentation. These advantages make it particularly suitable for rapid preliminary screening in primary healthcare settings, community clinics, or public health emergencies.

#### 3.3.2. Specificity

**Figure 4:**
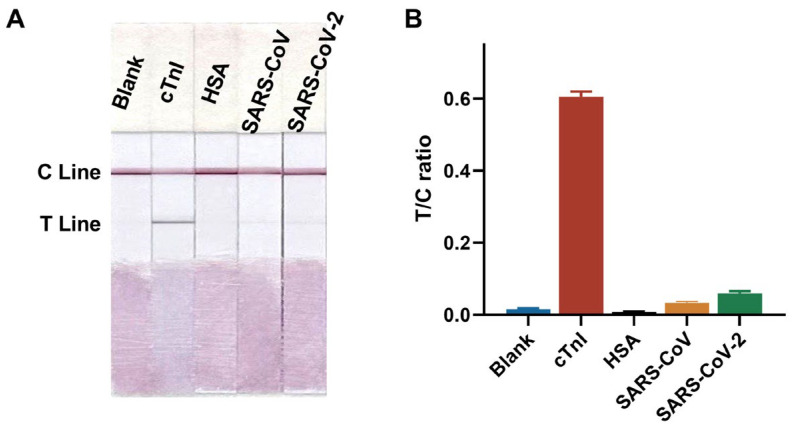
Specificity evaluation of the aptamer-based LFA for cTnI detection. (**A**) Visual LFA results for various targets, including cTnI, HSA, SARS-CoV, and SARS-CoV-2. (**B**) Quantitative analysis of T/C ratios corresponding to different targets, calculated using ImageJ.

To evaluate the specificity of the proposed aptamer-based LFA, potential cross-reactivity with structurally or clinically relevant interfering proteins was assessed. Specifically, human serum albumin (HSA), severe acute respiratory syndrome coronavirus (SARS-CoV), and SARS-CoV-2 were selected as representative non-target analytes. These analytes were each spiked at 1 μg·mL^−1^, along with cTnI as the positive target control. As shown in Figure 4A, only the sample containing cTnI produced a clearly visible red band at the T line, while no significant signals were observed in the HSA, SARS-CoV, SARS-CoV-2, or blank groups. Quantitative analysis of the T/C ratio further confirmed this observation: the cTnI group exhibited a significantly higher T/C ratio compared to all other groups (Figure 4B), demonstrating the excellent target specificity of the dual-aptamer LFA. Notably, SARS-CoV and SARS-CoV-2 proteins were included in the interference panel due to their potential co-occurrence in patients presenting with cardiac symptoms during viral infections, which could lead to diagnostic confusion or false positives. The absence of cross-reactivity in these groups underscores the assay’s high clinical specificity and robustness in complex biological matrices.

#### 3.3.3. Repeatability and Stability

**Figure 5:**
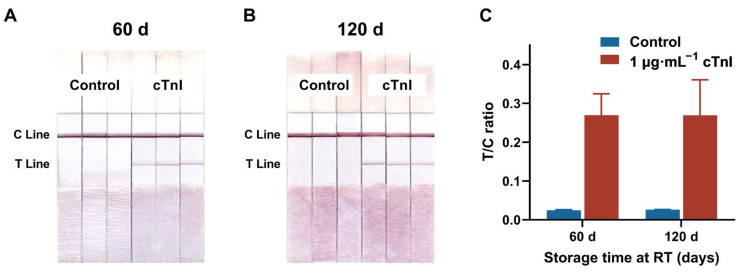
Stability evaluation of the aptamer-based LFA strips under RT storage. (**A**,**B**) Visual results of the LFA strips stored at RT for 60 days (**A**) and 120 days (**B**), followed by testing with blank buffer (Control) and 1 μg·mL^−1^ cTnI. (**C**) T/C ratio analysis of the test lines using ImageJ software. All data are presented as mean ± SD, n = 3.

The long-term stability of the aptamer-based LFA was assessed by storing the assembled strips at RT in sealed pouches for 60 and 120 days. The strips were subsequently used to detect 1 μg·mL^−1^ of cTnI under standard testing conditions. As shown in Figure 5A,B, the T lines for the cTnI groups maintained a clearly visible readout even after prolonged storage, whereas the control groups exhibited negligible background readout. Quantitative analysis of the T/C ratios (Figure 5C) demonstrated consistent signal intensities for both storage durations, indicating good repeatability and minimal performance degradation over time. These findings demonstrate that the fabricated LFA strips maintain both structural integrity and assay performance after long-term storage under RT conditions for at least four months, highlighting their applicability in point-of-care diagnostics in resource-limited settings.

It is worth noting that, in this study, a cTnI concentration of 1000 ng·mL^−1^—significantly higher than the typical clinical diagnostic range—was selected for long-term stability evaluation in order to minimize the impact of detection limit thresholds or visual judgment errors. As a result, the observed stability may not fully reflect the performance of the test strips at lower, clinically relevant concentrations. Future studies should further assess the storage stability of the test strips across a broader concentration range, particularly within the early diagnostic window of AMI, to further ensure the robustness and reliability of the assay in real-world clinical applications.

#### 3.3.4. Accuracy and Precision

To evaluate the analytical accuracy and precision of the aptamer-based LFA, recovery experiments were performed by spiking artificial serum samples with two concentrations of cTnI: 200 ng·mL^−1^ (low level) and 600 ng·mL^−1^ (high level). As summarized in Table 3, the intra-assay recoveries were 95.11% and 103.17%, while the inter-assay recoveries were 99.48% and 101.21%, respectively. The coefficients of variation (CVs) ranged from 1.32% to 3.94%, indicating good repeatability and precision. These results demonstrate that the proposed aptamer-based LFA achieves acceptable accuracy and precision for quantitative detection of cTnI in serum samples, meeting the analytical requirements for clinical biomarker analysis.

## 4. Conclusions

In this study, we developed a dual-aptamer sandwich-type LFA for the visual and semi-quantitative detection of cTnI, enabling rapid diagnosis of AMI without the need for complex instrumentation. The proposed aptamer-based LFA exhibited satisfactory analytical performance, achieving a detection limit of 11.70 ng·mL^−1^, along with excellent specificity, precision, and accuracy under optimized conditions. While the sensitivity is lower than that of some electrochemical strategies—such as an amperometric aptasensor with a reported LOD of 0.6 pg·mL^−1^ [15]—our method offers distinct advantages for POCT, including low cost, portability, instrument-free operation, and 10 min readout time. These features make it especially suitable for early AMI screening in resource-limited settings, such as primary care units, rural clinics, and emergency triage. Looking forward, further improvements to the aptamer-based LFA platform could focus on integrating isothermal nucleic acid amplification strategies such as hybridization chain reaction (HCR) or catalytic hairpin assembly (CHA) to enhance detection sensitivity without relying on enzymes or external power sources. Moreover, multiplex detection of cardiac biomarkers on a single strip, combined with novel signal reporters such as quantum dots and upconversion nanoparticles, may enable comprehensive cardiovascular risk profiling. Integration with microfluidic chips and smartphone-based image analysis could also realize fully automated, low-cost, and field-deployable platforms. These advancements are expected to drive the next generation of point-of-care diagnostics toward more sensitive, specific, and intelligent solutions for early AMI screening and cardiovascular disease management.

## Figures and Tables

**Table 1:** DNA sequences used in this study.

Name	DNA Sequence (5′ to 3′)	Modification
C line	AAAAAAAAAAAAAAAAAAAA	5′Biotin
T line	CGCATGCCAAACGTTGCCTCATAGTTCCCTCCCCGTGTCC	5′Biotin
Tro4	CGTGCAGTACGCCAACCTTTCTCATGCGCTGCCCCTCTTATTTTTTTTTTTTTTTTTTTT	3′SH C6

**Table 2:** Comparison of aptamer-based LFAs targeting various biomarkers and different types of cTnI detection biosensors.

Targets	Methods	LOD	Time	Reporter	Reference
Creatine kinase-MB	aptamer-based LFA	3.6 μg·mL^−1^	15 min	AuNPs	[27]
C-reactive protein	aptamer-based LFA	10 μg·mL^−1^	30 min	AuNPs	[28]
Deoxynivalenol	aptamer-based LFA	24.11 ng·mL^−1^	10 min	AuNPs	[29]
SARS-CoV-2	aptamer-based LFA	–	5–6 min	AuNPs	[30]
cTnI	aptamer-based LFA	11.70 ng·mL^−1^	10 min	AuNPs	This study
cTnI	ECL aptasensor	24 pg·mL^−1^	10 min	hydrazine	[25]
cTnI	colorimetric aptasensor	27 pg·mL^−1^	65 min	horseradish peroxidase	[11]
cTnI	ECL immunosensor	0.94 ng·mL^−1^	65 min	luminol	[31]
cTnI	antibody-based LFA	30 pg·mL^−1^	30 min	upconverting nanoparticle	[32]

**Table 3:** Accuracy and precision of the aptamer-based LFA for the detection of cTnI spiked in serum. All values are expressed as mean ± SD, n = 3.

cTnI (ng·mL^−1^)	Intra-Assay				Inter-Assay			
	Mean	SD	Recovery	CV (%)	Mean	SD	Recovery	CV (%)
200	190	7.49	95.11%	3.94	199	3.85	99.48%	1.93
600	619	8.17	103.17%	1.32	607	9.73	101.21%	1.60

## Data Availability

Dataset available on request from the authors.

## Supplementary Materials

The following supporting information can be downloaded at: https://www.mdpi.com/article/10.3390/bios15120776/s1, Figure S1: DLS analysis of bare AuNPs and AuNPs@Tro4.
