# Supplementary material for: Aptamer-Based Gold Nanoparticle Lateral Flow Assay for Rapid Detection of Cardiac Troponin I
Source: Biosensors (Basel). 2025 Nov 26;15(12):776. doi: 10.3390/bios15120776 (PMC12730940; doi:10.3390/bios15120776)
Supplement: Supplementary file 1 [file biosensors-15-00776-s001.zip › biosensors-3971315-supplementary.pdf]

biosensors

MDPI

Supplementary Materials

Aptamer-Based Gold Nanoparticle Lateral Flow Assay for Rapid Detection of Cardiac Troponin I

Jing Zhang [1,*], Jiayi Pang [1] and Cheng Cui [2,*]

[1] School of Biological Science and Medical Engineering, Hunan University of Technology, Zhuzhou 412007, China
[2] Molecular Science and Biomedicine Laboratory (MBL), State Key Laboratory of Chemo and Biosensing, College of Chemistry and Chemical Engineering, College of Biology, Aptamer Engineering Center of Hunan Province, Hunan University, Changsha 410082, China
* Correspondence: zhang_jing@hut.edu.cn (J.Z.); ccui@hnu.edu.cn (C.C.)

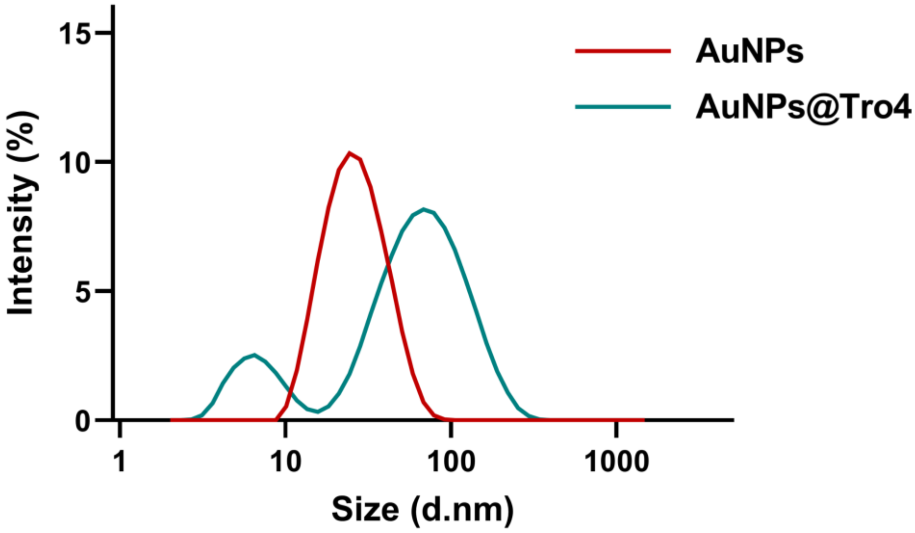

Figure S1. DLS analysis of bare AuNPs and AuNPs@Tro4. The hydrodynamic diameter increased from approximately 22 nm (red solid line) to 65 nm (green solid line) after conjugation with the thiolated aptamer Tro4, indicating successful surface modification. The absence of large-size peaks suggests that the conjugates remained colloidally stable without significant aggregation.
